# Supplementary material for: Drug repurposing for aging research using model organisms
Source: Aging Cell. 2017 Jun 16;16(5):1006–15. doi: 10.1111/acel.12626 (PMC5595691; doi:10.1111/acel.12626)
Supplement: Supplementary file 7 — Data S1 Zip‐Archive of all report cards. [file ACEL-16-1006-s007.zip › RC_RAP.pdf]

RAP

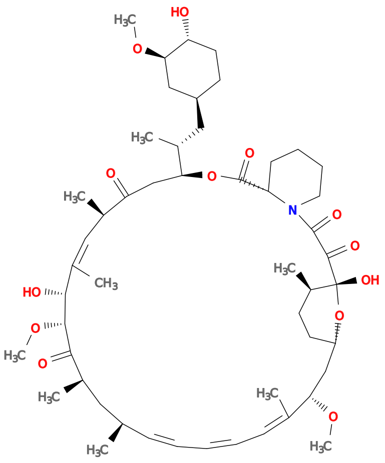

Database identifiers

ChEMBLCompound	CHEMBL413
CHEBI	9168
eMolecules	30264609
eMolecules	31590036
eMolecules	36500353
eMolecules	594011

Ranking

	Rank	Score
Drosophila	95/697	0.792
C. elegans	357/591	0.166

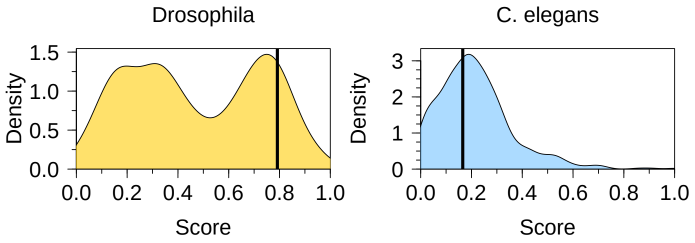

	Ageing implication	Domain conservation	Binding site conservation	Binding affinity	Bioavailability	Lipinski	Promiscuity	Purchasability	Drug approval	**Total**
Drosophila	1.0	0.967	0.921	0.926	(0.9)	-0.15	-0.0	0.1	0.1	0.792
C. elegans	1.0	0.927	0.675	0.926	0.2	-0.15	-0.0	0.1	0.1	0.166

Names

- **sirolimus**
- (1R,9S,12S,15R,16E,18R,19R,21R,23S,24E,26E,28E,30S,32S,35R)-1,18-dihydroxy-12-(2S)-1-(1S,3R,4R)-4-hydroxy-3-methoxycyclohexylpropan-2-yl-19,30-dimethoxy-15,17,21,23,29,35-hexamethyl-11,36-dioxa-4-azatricyclo30.3.1.0(4,9)hexatriaconta-16,24,26,28-tetraene-2,3,10,14,20-pentone
- Antibiotic AY 22989
- Rapamune
- Rapamycin

Roles

immunosuppressive agent, antineoplastic agent, antibacterial drug, bacterial metabolite, mTOR inhibitor, FK506-binding protein 1A inhibitor

Status

Approved drug (according to ChEMBL)	Yes
Number of Rule of 5 violations	3
Binding affinity to original target in log units (RF-Score prediction)	7.53
Burns *C. elegans* bioavailability prediction	-13.99

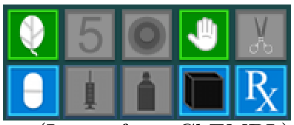

(Image from ChEMBL)

Compound Target Characteristics

Serine/threonine-protein kinase mTOR

Best gene implication in ageing for this target family came from gene Q9VK45 annotated in UniProt release 2014_02. Annotation GO 8340 (determination of adult lifespan) was Inferred from Mutant Phenotype

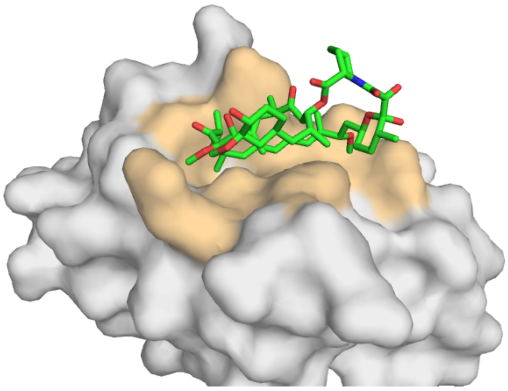

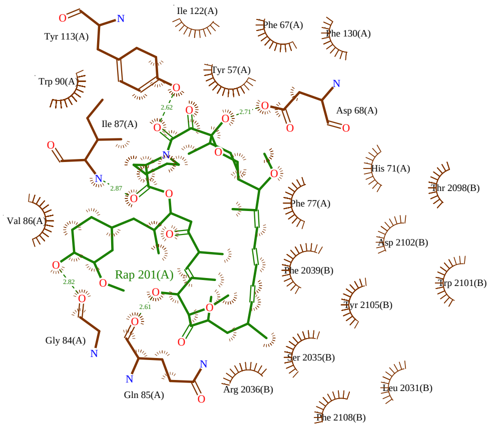

protein	amino acids contacts (binding site)									
PDB:1fap:chainB:P42345	L	E	S	R	F	T	W	D	Y	F
PDB:4drh:chainB:P42345	L	E	S	R	F	T	W	D	Y	F
PDB:4drh:chainE:P42345	L	E	S	R	F	T	W	D	Y	F
PDB:4dri:chainB:P42345	L	E	S	R	F	T	W	D	Y	F
PDB:4drj:chainB:P42345	L	E	S	R	F	T	W	D	Y	F
tr:B1AKP8:B1AKP8_HUMAN	L	E	S	R	F	T	W	D	Y	F
tr:Q96QW8:Q96QW8_HUMAN	L	E	S	R	F	T	W	D	Y	F
sp:P42345:MTOR_HUMAN	L	E	S	R	F	T	W	D	Y	F
sp:P42346:MTOR_RAT	L	E	S	R	F	T	W	D	Y	F
sp:Q9JLN9:MTOR_MOUSE	L	E	S	R	F	T	W	D	Y	F
tr:Q960H0:Q960H0_DROME	L	E	S	R	F	D	W	D	Y	F
tr:H1ZYC1:H1ZYC1_DROME	L	E	S	R	F	D	W	D	Y	F
sp:Q9VK45:TOR_DROME	L	E	S	R	F	D	W	D	Y	F
tr:H1ZYB7:H1ZYB7_DROME	L	E	S	R	F	D	W	D	Y	F
tr:H1ZYB5:H1ZYB5_DROME	L	E	S	R	F	D	W	D	Y	F
sp:Q95Q95:TOR_CAEEL	L	D	S	R	F	N	W	E	C	F
sp:P32600:TOR2_YEAST	L	D	S	R	F	N	W	D	Y	F

protein	whole protein ident	simil	domain-based ident	simil	contact-based ident	simil
PDB:1fap:chainB:P42345	1.0	1.0	1.0	1.0	1.0	1.0
PDB:4drh:chainB:P42345	0.93	0.97	0.94	0.97	1.0	1.0
PDB:4drh:chainE:P42345	0.93	0.97	0.93	0.97	1.0	1.0
PDB:4dri:chainB:P42345	0.92	0.96	0.94	0.97	1.0	1.0
PDB:4drj:chainB:P42345	0.94	0.98	0.94	0.98	1.0	1.0
tr:B1AKP8:B1AKP8_HUMAN	0.3	0.3	1.0	1.0	1.0	1.0
tr:Q96QW8:Q96QW8_HUMAN	0.35	0.35	1.0	1.0	1.0	1.0
sp:P42345:MTOR_HUMAN	1.0	1.0	1.0	1.0	1.0	1.0
sp:P42346:MTOR_RAT	0.99	1.0	1.0	1.0	1.0	1.0
sp:Q9JLN9:MTOR_MOUSE	0.99	1.0	1.0	1.0	1.0	1.0
tr:Q960H0:Q960H0_DROME	0.2	0.26	0.77	0.94	0.9	0.92
tr:H1ZYC1:H1ZYC1_DROME	0.51	0.8	0.77	0.94	0.9	0.92
sp:Q9VK45:TOR_DROME	0.51	0.8	0.77	0.94	0.9	0.92
tr:H1ZYB7:H1ZYB7_DROME	0.51	0.8	0.77	0.94	0.9	0.92
tr:H1ZYB5:H1ZYB5_DROME	0.51	0.8	0.77	0.94	0.9	0.92
sp:Q95Q95:TOR_CAEEL	0.32	0.67	0.58	0.85	0.6	0.68
sp:P32600:TOR2_YEAST	0.39	0.72	0.59	0.87	0.8	0.9

Tor (FBgn0021796) associated phenotypes

body size defective, cell cycle defective, cell size defective, chemical sensitive, decreased cell number, decreased cell size, developmental rate defective, dominant, germline clone, gravitaxis defective, increased cell death, increased cell size, lethal - all die before end of pupal stage, lethal - all die before end of second instar larval stage, long lived, mitotic cell cycle defective, neuroanatomy defective, nutrition conditional, size defective, small body, somatic clone, some die during pupal stage, some die during second instar larval stage, some die during third instar larval stage, sterile

(Information from FlyBase)

Tor (UniProt:Q9VK45) annotation

Function: Promotes cell and tissue growth, maintains tissue homeostatis and controls responses to environmental stress and aging. Regulates growth during animal development by coupling growth factor signaling to nutrient availability. Central regulators of autophagy. May be involved in atg1 phosphorylation. May also be involved, directly or indirectly, in the control of neuronal function. Phosphorylates S6K/p70S6K, in vitro. May regulate the activity of S6K. Overexpression inhibits growth and reduces cell size. Affects the timing of neuronal cell differentiation. Hyperactivation of the signaling leads to accelerated differentiation, whereas inhibition of the signaling retards differentiation. Thus, in addition to controlling growth of the cell in which it resides, it can also influence growth of distant cells and organs during development via a humoral mechanism. In short, during development, it primarily controls growth, whereas in the adult, where there is relatively little growth, it controls aging and other aspects of nutrient-related physiology. Rag GTPases act as activators of TORC1 in response to amino acid signals. (PubMed:11069885, PubMed:11069888, PubMed:12559758, PubMed:14505573, PubMed:15454083, PubMed:16469695, PubMed:18604198, PubMed:19211682, PubMed:19225150).
Subunit: May be part of a minimal complex, TORC1, consisting oftor, raptor and lst8. May be part of a minimal complex, TORC2, consisting of tor, rictor and lst8 (By similarity). Self-associates; assembles into homomultimeric complexes. Component of a multiprotein complex. (, PubMed:16219781).
Induction: By PI3K/Akt signaling, or by nutrients such as amino acids, and by high cellular energy levels. (PubMed:19211682).
Disruption phenotype: Not lethal. Displays phenotypes characteristic of amino acid deprivation, including reduced nucleolar size, lipid vesicle aggregation in the larval fat body, and a cell type-specific pattern of cell cycle arrest that can be bypassed by overexpression of the S-phase regulator cyclin E. Reach only the size of second instar larvae, at which point they undergo cell cycle arrest. (PubMed:11069885, PubMed:11069888).

(Information from UniProt)

let-363 (WBGene00002583) associated phenotypes
adult growth variant, late larval lethal, lethal

(Information from WormBase)

let-363 (UniProt:Q95Q95) annotation
Function: Kinase that regulates the mRNA translation machinery, probably by modulating the activity of translation factors such as eIF-4G and eIF-2. It may have some protein kinase activity instead of lipid kinase activity. May act as a mediator of lifespan regulation by insulin signaling and nutrient sensing. (PubMed:12225660, PubMed:14668850).
Subcellular location: Nucleus (PubMed:12225660).
Tissue specificity: Ubiquitous. Expressed in all major tissues and organs, including the intestine, gonads and hypodermal cells. (PubMed:12225660).
Disruption phenotype: Defects strongly increase lifespan, the mean lifetime being of 25 days instead of 10 days, suggesting that it may be involved in aging process. (PubMed:14668850).

(Information from UniProt)
